# Left Gastric Artery Pseudoaneurysm Complicating Chronic Calcifying Pancreatitis in a Child

**DOI:** 10.7759/cureus.34073

**Published:** 2023-01-23

**Authors:** Md S Ahmad, Biswajit Sahoo, Kanishka Das, Akash Pati

**Affiliations:** 1 Pediatric Surgery, All India Institute of Medical Sciences, Bhubaneswar, Bhubaneswar, IND; 2 Radiology, All India Institute of Medical Sciences, Bhubaneswar, Bhubaneswar, IND

**Keywords:** interventional radiology guided embolization, vascular complication, left gastric artery embolization, treatment of chronic pancreatitis, artery pseudoaneurysm

## Abstract

A left gastric artery pseudoaneurysm is a rare complication of pancreatitis and is associated with significant morbidity and mortality. We report a 14-year-old male with severe abdominal pain and a palpable upper abdominal mass, earlier diagnosed as chronic idiopathic calcifying pancreatitis, and awaiting surgical intervention. Computed tomography showed a pseudocyst and a pseudoaneurysm in the lesser sac near the left gastric artery. The patient underwent successful angiographic coiling of the left gastric artery and definitive pancreatic surgery weeks thereafter. The early detection and interventional radiologic management of the vascular complication averted a life-threatening hemorrhage without emergency surgery in a pediatric patient.

## Introduction

A vascular complication of chronic pancreatitis is rarely reported in children [1,2]. It commonly occurs in the spleno-porto-mesenteric vessels as thrombosis or pseudoaneurysms, and the splenic vein is commonly involved. Pseudoaneurysms associated with pancreatitis are common in patients with pseudocysts although they also occur without them [3]. The rupture of an aneurysm into a pseudocyst, gastrointestinal tract, or peritoneal cavity is dreadful. It is difficult to differentiate between a pseudoaneurysm and a bleeding pseudocyst [4]. Unlike a true aneurysm, a pseudoaneurysm lacks all the layers of the vessel and hence is more liable to bleed. It can bleed through the ampulla of Vater, known as hemosuccus pancreaticus, or into adjacent organs following fistulization. Although emergency surgery to control the hemorrhage is opted for in unstable patients, transcatheter embolization can be attempted in select hemodynamically stable patients. We describe a patient with a left gastric artery aneurysm successfully managed by selective artery embolization. 

## Case presentation

**Figure 1:**
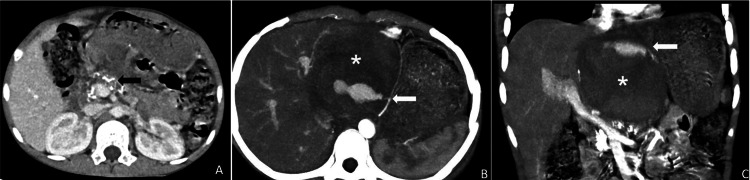
CT scan A: CECT axial venous phase showing chronic calcific pancreatitis (black arrow); B: Axial and C: Coronal maximum intensity projection arterial phase images showing hyperdense content (blood /clots) within the pseudocyst (asterisk) with evidence of pseudoaneurysm (white arrow) arising from the left gastric artery.

A 14-year-old male with idiopathic chronic calcific pancreatitis presented to the emergency department with severe abdominal pain. He had been extensively investigated earlier and diagnosed with idiopathic obstructive pancreatitis. Serum biochemistry comprising calcium, phosphorous, alkaline phosphatase, and lipid profile were normal. Anatomic evaluation for structural congenital anomalies of the biliary pancreatic tree was not in evidence. He was on a low-fat diet, pancreatic enzyme supplementation, and maintenance analgesics and awaited elective pancreatoenteric diversion. There was evidence of chronic calcific pancreatitis with predominant head affliction, intraductal calculi, and obstructed dilated duct all along the length of the pancreas from neck to tail. However, there was no pseudocyst formation, which was noted when the child presented with an acute abdomen and pallor four weeks thereafter while waiting for elective surgery. At presentation, there was no history of vomiting, diarrhea, hematemesis, or melena. He was tachycardic (116/min) and hypotensive (blood pressure 96/68 mmHg) at admission. A smooth, tender mass of 6 cm X 8 cm was palpable in the epigastric region extending to both hypochondriac regions. The hemoglobin was 3.5 g/dL, and serum amylase/lipase was normal. The resuscitation after the presentation as acute abdomen and pallor and a diagnosis of a pseudocyst with pseudoaneurysm included fluid resuscitation (crystalloids, packed cell transfusion), empiric IV antibiotics (piperacillin-tazobactam, metronidazole), pantoprazole, and hemodynamic monitoring with central venous line and bladder catheter till stability was obtained by 48 hours. It was then that a considered decision was made by the team to embolize the aneurysm with the operating theater as a standby in case the procedure fails and an emergency laparotomy is required. He responded well to resuscitation and was hemodynamically stable after initial resuscitation. The contrast-enhanced computed tomography (CECT) showed features of chronic calcific pancreatitis with a 5x6 cm pseudocyst in the lesser sac (Figure 1A). The pseudocyst was filled with hyperdense content (blood products/clots) associated with a pseudoaneurysm from the left gastric artery (Figures 1B, 1C).

**Figure 2:**
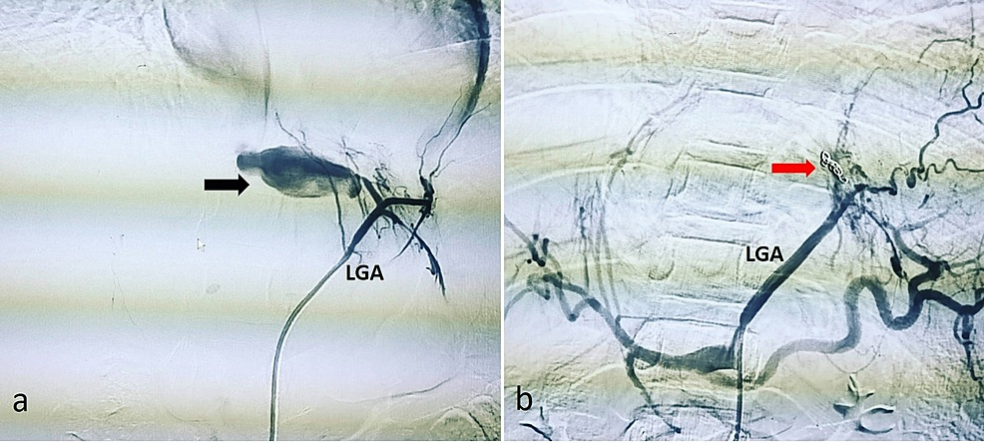
Digital subtraction angiography (a) Digital subtraction angiography (DSA) showing a pseudoaneurysm (arrow) arising from a first-order branch of the left gastric artery (LGA); (b) DSA showing a completely knocked-off first-order branch of the left gastric artery (LGA) without filling up of the pseudoaneurysm. The arrow indicates embolizing coils.

The operation theatre was kept ready, and an immediate digital subtraction angiography (DSA) was performed. It confirmed a pseudoaneurysm in the left gastric artery (Figure 2a). A microcatheter was navigated into the culprit branch of the left gastric artery, and two coils were deployed therein. A post-embolization run showed no active contrast extravasation into the pseudoaneurysm (Figure 2b).

After the embolization procedure, the patient was closely followed up until stability and discharged on a normal diet after seven days. After six weeks, he underwent a Frey’s procedure. A routine ultrasonogram imaging was performed before surgery to confirm the resolution of any peripancreatic collection. The patient was regularly followed up for the resolution of symptoms clinically and radiologically with USG imaging. At the one-year follow-up, he was asymptomatic and well without any endocrine dysfunction.

## Discussion

Visceral intra-abdominal pseudoaneurysms are rare, most commonly involving the splenic artery (60%), followed by the hepatic artery (20%), superior mesenteric artery (5%), and celiac trunk (4%) [4]. The splenic vessels have maximal proximity to the pancreas; coursing on the pancreatic bed, they are most likely to be affected by the pancreatic enzymes. Rarely, it involves the left gastric artery and is usually secondary to an adjacent inflammatory process [5]. Unlike adults, the absence of predisposing factors like atherosclerosis and hypertension makes it rarer [6]. Chronic local inflammation leads to an increased local elastase with autodigestion of peripancreatic vessels or erosion of a concomitant pseudocyst into the artery [7]. Autodigestion of the pancreatic vessels leads to pseudoaneurysm formation, which may rupture and bleed into the pseudocyst [8]. The patients present acutely with abdominal pain, and a rupture of the pseudoaneurysm can lead to shock [5]. Arising from the coeliac axis, the left gastric artery turns sharply and traverses on the lesser curvature of the stomach. Pseudoaneurysms can arise in the section of the artery lying either outside the gastric wall (extramural) or inside the gastric wall (intramural). Rupture of pseudoaneurysm can lead to life-threatening hemorrhage into the stomach, peritoneal cavity, or pseudocyst [9].

Ultrasound with Doppler could image the pseudocysts or pseudoaneurysms of the peripancreatic artery. The pseudoaneurysms have internal hemodynamics like high-velocity central jets with low flow adjacent to the walls, which can be detected by color Doppler ultrasound. Color Doppler sonography can also evaluate complex flow patterns such as bidirectional or swirling flow and a mosaic color configuration [10]. Computed tomography may identify the culprit pseudoaneurysm or pseudocyst and demonstrate active bleeding. However, selective visceral angiography remains the gold standard for diagnosis and therapy.

Interventional radiological procedures and surgery are the main treatment modalities. Visceral artery aneurysms require surgical management when they are symptomatic and/or more than two cm in diameter [11]. If the patient is hemodynamically stable, endovascular procedures (coil or glue embolization) offer an alternative to conventional open surgery with the advantage of low procedural morbidity and mortality [12]. In hemodynamically unstable patients, emergency procedures are inevitable and include ligation of the feeding vessels or resection of the aneurysm with various arterial reconstructions [11,13]. In a retrospective study, Carr et al. reported less morbidity in stable patients undergoing angioembolization than in surgery [14]. The rebleeding rates are reported to be around 15% following transcatheter angioembolization [15]. Failed embolization or rebleed can be managed by directly ligating the bleeding vessel or resecting the pancreas with a pseudoaneurysm [16].

## Conclusions

A pancreatic pseudoaneurysm can lead to a life-threatening complication. Pseudoaneurysm of the left gastric artery in chronic pancreatitis is rare, especially in children. In select stable patients, an emergency digital subtraction angiography and successful embolization of the precise vessel are feasible. It can prevent a life-threatening complication and avoid emergency surgical management with its associated morbidity and mortality.
